# Combining bulk RNA-sequencing and single-cell RNA-sequencing data to reveal the immune microenvironment and metabolic pattern of osteosarcoma

**DOI:** 10.3389/fgene.2022.976990

**Published:** 2022-10-19

**Authors:** Ruichao Huang, Xiaohu Wang, Xiangyun Yin, Yaqi Zhou, Jiansheng Sun, Zhongxiu Yin, Zhi Zhu

**Affiliations:** ^1^ Department of Orthopedics, Zhengzhou Central Hospital affiliated to Zhengzhou University, Zhengzhou, China; ^2^ Advanced Medical Research Center of Zhengzhou University, Zhengzhou Central Hospital affiliated to Zhengzhou University, Zhengzhou, China; ^3^ Nanchang University Queen Mary School, Nanchang, China

**Keywords:** osteosarcoma, metabolism, bulk RNA sequencing, single cell RNA sequencing, tumor microenvironment

## Abstract

**Background:** Osteosarcoma (OS) is a kind of solid tumor with high heterogeneity at tumor microenvironment (TME), genome and transcriptome level. In view of the regulatory effect of metabolism on TME, this study was based on four metabolic models to explore the intertumoral heterogeneity of OS at the RNA sequencing (RNA-seq) level and the intratumoral heterogeneity of OS at the bulk RNA-seq and single cell RNA-seq (scRNA-seq) level.

**Methods:** The GSVA package was used for single-sample gene set enrichment analysis (ssGSEA) analysis to obtain a glycolysis, pentose phosphate pathway (PPP), fatty acid oxidation (FAO) and glutaminolysis gene sets score. ConsensusClusterPlus was employed to cluster OS samples downloaded from the Target database. The scRNA-seq and bulk RNA-seq data of immune cells from GSE162454 dataset were analyzed to identify the subsets and types of immune cells in OS. Malignant cells and non-malignant cells were distinguished by large-scale chromosomal copy number variation. The correlations of metabolic molecular subtypes and immune cell types with four metabolic patterns, hypoxia and angiogenesis were determined by Pearson correlation analysis.

**Results:** Two metabolism-related molecular subtypes of OS, cluster 1 and cluster 2, were identified. Cluster 2 was associated with poor prognosis of OS, active glycolysis, FAO, glutaminolysis, and bad TME. The identified 28608 immune cells were divided into 15 separate clusters covering 6 types of immune cells. The enrichment scores of 5 kinds of immune cells in cluster-1 and cluster-2 were significantly different. And five kinds of immune cells were significantly correlated with four metabolic modes, hypoxia and angiogenesis. Of the 28,608 immune cells, 7617 were malignant cells. The four metabolic patterns of malignant cells were significantly positively correlated with hypoxia and negatively correlated with angiogenesis.

**Conclusion:** We used RNA-seq to reveal two molecular subtypes of OS with prognosis, metabolic pattern and TME, and determined the composition and metabolic heterogeneity of immune cells in OS tumor by bulk RNA-seq and single-cell RNA-seq.

## Introduction

Osteosarcoma (OS) is an aggressive osteoid-producing tumor of mesenchymal origin, characterized by a complex and, frequently, uncertain etiology (Mercatelli et al., 2018). It is widely believed that the etiology of OS contains epidemiologic factors, genetic impairments and environmental factors. Presently, recognized risk factors related to the progress of osteosarcoma consist of Paget’s disease, hereditary retinoblastoma, other chromosomal abnormalities, ionizing radiation, and alkylating agents (Jafari et al., 2020). Chemotherapy, followed by total surgical resection and then post-operative adjuvant chemotherapy as well as radiotherapy, is currently the standard treatment strategy for OS (Xie et al., 2022). Whereas, traditional surgical resection combined with chemotherapy has many limitations, such as drug resistance and systemic side effects of chemotherapeutic drugs, postoperative recurrence, bone defect and so on (Wu et al., 2022). The overall survival rate of local OS is more than 70%, while the survival rate of metastatic, refractory and recurrent osteosarcoma is low (Yang et al., 2021). Especially metastatic OS, the proportion of patients with long-term survivors is 20–30% (Leite et al., 2021). It is reported that for patients with metastatic diseases, especially those with chemotherapy-resistant/refractory diseases, DNA and RNA analysis are generally considered to provide information about further potential therapeutic targets (Smrke et al., 2021). And because osteosarcoma is one of the most heterogeneous cancer entities in human beings. This heterogeneity occurs not only at the macro and micro levels with heterogeneous microenvironmental components, but also at the genome, transcriptome and epigenetic levels (Schiavone et al., 2019). Therefore, from these aspects to understand the heterogeneity amongst osteosarcomas may be key to improve patient outcomes.

Recent studies have shown that metabolic abnormalities are a major marker of cancer. Tumor metabolism shows unique behavior and plays an important role in tumor growth and metastasis, making it an attractive potential target for new therapy (Leite et al., 2021). Similar to genetic heterogeneity, the metabolic phenotype of cancer is highly heterogeneous. Abnormal metabolic phenotypes of cancer, such as aerobic glycolysis, pentose phosphate pathway (PPP), increased glutamine metabolism and fatty acid oxidation (FAO), are important factors leading to tumor malignancy, metastasis and drug resistance, which are significantly affected by cancer subtypes and specific tumor microenvironment (TME) (Park et al., 2020). Unraveling the complexity of how genetics, microenvironment and genes interact to produce metabolic dependence will be a challenge, but may provide a path to exploit metabolism in a way that could be transformative for patients (Luengo et al., 2017).

Sequencing technology provides tools for revealing the complex interactions of tumor metabolism, microenvironment and genes. Bulk RNA sequencing (bulk RNA-seq) and single-cell RNA sequencing (scRNA-seq) are the application of mainstream sequencing technology at present (Li et al., 2021). Bulk RNA-seq is the most widely used genomic technique for studying the transcriptional landscape and altered molecular pathways in human cancers, which only provides the average gene expression profiles in different cell clusters and cannot capture the transcriptional heterogeneity prevalent in cell populations (Wang et al., 2020). Compared with bulk RNA-seq, scRNA-seq provides high-throughput and high-resolution transcriptome profiling of individual cells, generating much noisier and more variable data (Chen et al., 2019). ScRNA-seq can reveal the state and function of single cells by isolating single cells, capturing their transcripts, and generating sequencing libraries at the single-cell level (Ding et al., 2020). In this study, combined with these two sequencing techniques and RNA-seq, we aimed to classify OS based on metabolism-related genes and revealed the TME and metabolic heterogeneity of OS at the single cell level. This will help to understand the interaction among gene, TME and metabolism, and is expected to provide a meaningful theoretical basis for targeted metabolic therapy in patients with OS.

## Materials and methods

### Clinical data processing of osteosarcoma samples obtained from public data sets

The transcriptome and public clinical phenotypic data of osteosarcoma were downloaded from Target database, and a total of 79 tumor samples were obtained. The expression information of 24998 genes from 45 tumor samples, 20818 genes from 36 tumor samples and 13515 genes from 28 tumor samples were downloaded from Gene Expression Omnibus (GEO, http://www.ncbi.nlm.nih.gov/geo/) database with GSE21257, GSE3905 and GSE16091 as entry numbers, respectively.

### Download of metabolism-related pathways and single-sample gene set enrichment analysis

To investigate the metabolism-related molecular characteristics and pathways, a total of 76 genes related to the glycolysis, PPP and FAO and glutaminolysis processes were downloaded from Molecular Signatures Database (MSigDB, https://www.gsea-msigdb.org/gsea/index.jsp). There were 36 genes in glycolysis gene set, 22 genes related to PPP, 11 genes belong to FAO gene set, and 7 genes belong to glutaminolysis gene set. The removeBatchEffect function of limma and sva package was employed to remove the osteosarcoma data in batches, and the normalizeBetweenArrays function was utilized to correct the data set after the batch was removed. Single sample GSEA (ssGSEA) analysis was performed using GSVA software package to obtain the scores of the samples in four gene sets.

### ConsensusClusterPlus was conducted to identify metabolic subtypes

We used the “ConsensusClusterPlus” program to cluster osteosarcoma samples based on the sample scores of the four selected metabolic pathways. In this program, “hc” algorithm was employed to perform unsupervised clustering with “pearman” as the metric distance, which was performed 500 times with 80% of the total samples taken each time. The most suitable number of clusters was determined by cumulative distribution function (CDF) and consensus matrices.

### Evaluation of immune cell infiltration and important immune scores

The scores of 22 immune cells in osteosarcoma samples were estimated based on CIBERSORT algorithm. Important TME scores between different metabolic subtypes, including stomal score, immune score, ESTIMATE score, Toll-like receptor score, natural killer (NK) cytotoxicity score, antigen processing and presentation score, interferon-gamma (IFN-γ) score, cytolytic (CYT) activity score. Among them, stomal score, immune score and ESTIMATE score were calculated by the ESTIMATE algorithm. Toll-like receptor score, NK cytotoxicity score, antigen processing and presentation score were calculated using ssGSEA method based on the relevant genes of toll-like receptor signaling pathway, NK cell mediated cytotoxicity, and antigen processing and presentation pathway downloaded from MSigDB of GSEA.

### Analysis of differentially expressed genes of metabolic subtypes

The differentially expressed genes between metabolic subtypes were analyzed by limma toolkit, and the genes satisfying the requirements of | log2 (Fold Change) | > log2 (1.2) and *p* < 0.05 were defined as differentially expressed genes (DEGs) among metabolic subtypes. The intersections of DEGs between all data sets were taken, and the protein-protein interaction (PPI) network was analyzed by STRING (https://cn.string-db.org/), and the internal relationship between DEGs was visualized using Cytoscape. The Analyze Network of Cytoscape was used to calculate the degree in PPI network, and degree was used as the identification index of key nodes.

### Clustering dimension reduction of scRNA-seq data

In this study, the sequencing data of 50,174 cells from 6 tumor tissues from GSE162454 data sets were obtained, and the cell cluster analysis was carried out by Seurat package. The PercentageFeatureSet function in the Seurat package was applied to calculate the percentage of mitochondria and rRNA. Single cells with more than 35% mitochondria and less than 1000 UMI were removed. We normalized the data using log-normalization and identified top 2000 highly variable genes using FindVariableFeatures function. The principal component analysis (PCA) and t-distributed stochastic neighbor embedding (t-SNE) methods were used to reduce dimension and identify clustering.

### Estimation of cell proportion

The cell proportions were estimated using MuSiC((Wang et al., 2019)), which used a deconvolution method based on marker genes of cell types and gene expression matrices of both scRNA-seq and bulk RNA-seq to estimate the cell proportions of bulk RNA-seq data. The count-based expression data of both scRNA-seq and bulk RNA-seq was applied to this analysis.

### Functional enrichment analysis

The FindAllMarkers function identified significant marker genes using the threshold of log2 [Foldchange (FC)] = 0.5 and *p* < 0.05 adjusted by Minpct = 0.35 and Benjamini-Hochbergch procedure. And the significant marker genes were loaded onto the clusterProfiler for Encyclopedia of genes and Genomes pathway enrichment analysis.

### Statistical analysis

All the statistical analyses of this study were performed in R (version 4.1.0). Wilcoxon rank sum test was exploited to test the relationship of continuous variables between the two groups. *p*-value < 0.05 was defined as statistically significant.

## Results

### Identification of different metabolic molecular subtypes in osteosarcoma

**FIGURE 1:**
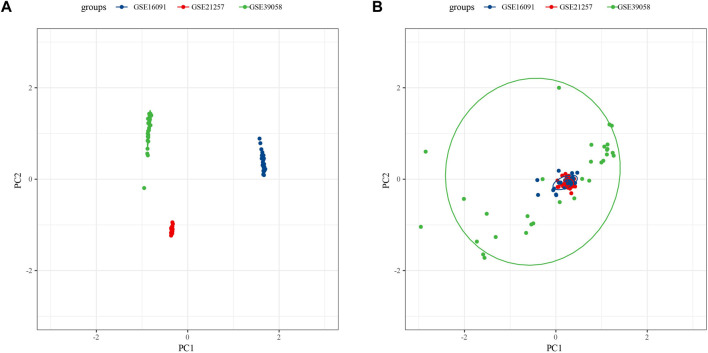
Osteosarcoma samples before eliminating the batch effect **(A)**, the clusters of different databases were clustered more closer than after removal **(B)**.

**FIGURE 2:**
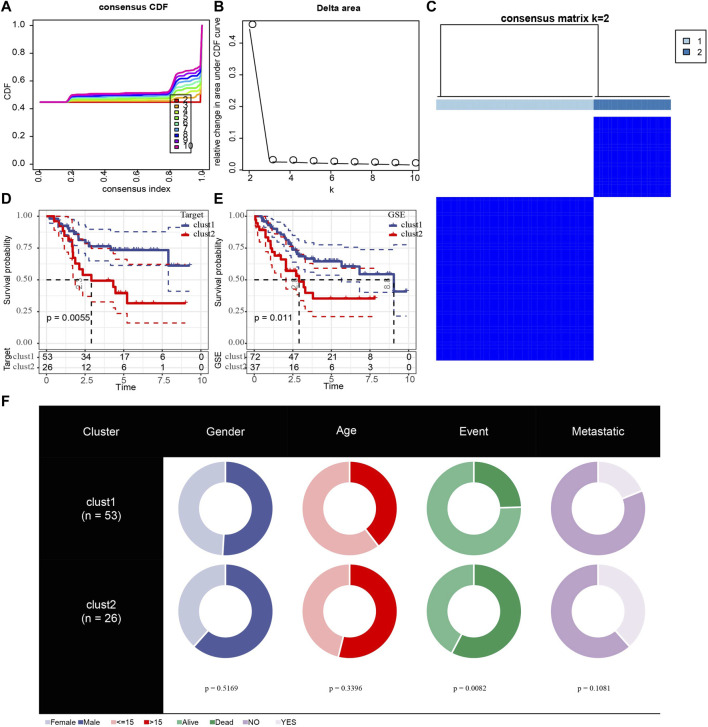
Identification of different metabolic molecular subtypes in osteosarcoma. **(A)**: The CDF curves of k at 2–10:00, respectively. **(B)**: Delta area curve of consensus clustering. **(C)**: consensus matrices for k = 2. **(D)**: The Kaplan Meier (KM) curve of cluster 1 and cluster 2 in the Target dataset. **(E)**: Survival analysis of two metabolic subtypes in merged GSE. **(F)**: Comparisons of clinicopathological features between cluster1 and cluster 2.

Because of clustering and batch effect removal are interrelated, the ideal batch effect removal method should be performed together with clustering (Li et al., 2020). Therefore, we first eliminated the batch effect in the three osteosarcoma data sets. It could be observed from PCA that the clusters of different databases were clustered more closer than after removal before the batch effect was removed (Figure 1). Then consensus clustering analysis was performed according to the scores of the four metabolic pathways. The obtained CDF and delta area curve of consensus clustering showed that the CDF value was the most stable and achieved adequate selection when k = 2 (Figures 2A–C). And PCA based on four metabolic pathways supported metabolic heterogeneity among tumors and the reliability of classifying OS into two metabolic subtypes (Supplementary Figure S1). Whether in the Target dataset or the merged GSE dataset, the prognosis of cluster 1 was significantly better than that of cluster 2 (Figures 2D,E). In addition, several clinicopathological features between cluster1 and cluster2 were compared. Different sex ratio, age distribution, survival status and metastasis ratio were observed in the two subtypes, but the differences were only observed in survival status, and the mortality rate in cluster 2 was significantly higher than that in cluster 1 (Figure 2F).

### Potential metabolic patterns of two subtypes

**FIGURE 3:**
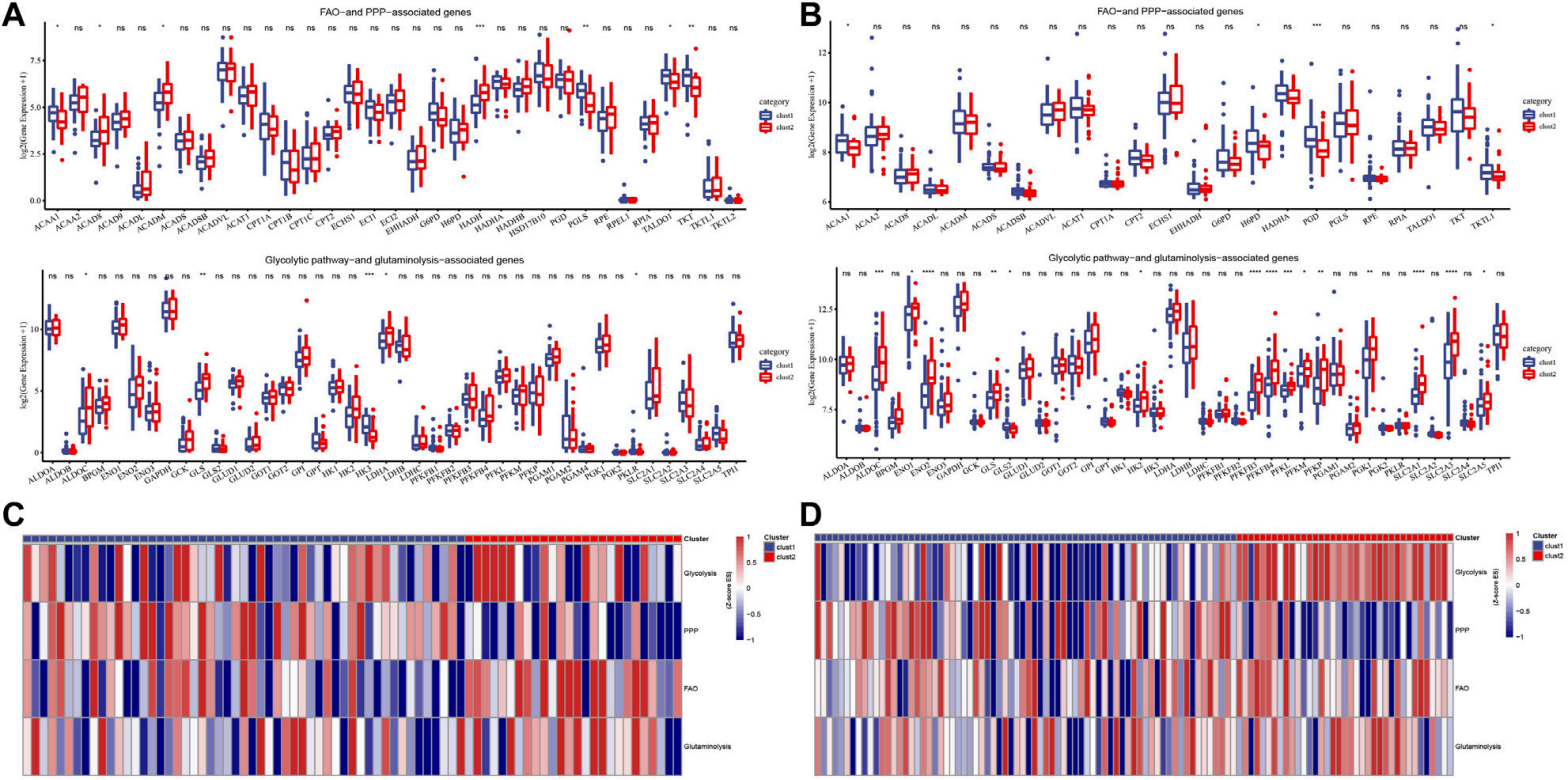
Potential metabolic patterns of two subtypes. **(A)**: The expression levels of genes in the four selected metabolic pathways between the two subtypes of the Target dataset. **(B)**: The differential expression analysis of four metabolism-related genes between cluster1 and cluster2 in the combined GSE dataset. **(C)**: The heat map shows the scores of different clusters of the Target dataset in the four metabolic pathways. **(D)**: Heat map of score trends of the two subtypes in four metabolic pathways in the combined GSE dataset. For this figure, the asterisks idicated the statistical *p* value, *p < 0.05, **p < 0.01, ***p < 0.001, ****p <0.0001.

To explore the metabolic patterns of the two subtypes in the Target dataset and the merged GSE dataset, the expression of genes in four selected metabolic pathways in the two subtypes was analyzed. Among the FAO and PPP related genes, ACAA1, ACAD8, ACADM, HADH and PGLS, TALDO1 and TKT had significant differences between the two subtypes. In all selected glycolytic pathway-and glutaminolysis-associated genes, ALDOC, GLS, HK3, LDHA and PKLR showed significant differences in expression between cluster 1 and cluster 2 (Figure 3A). The expression trends of ACAA1, H6PD, PGD, TKTL1, ALDOC, ENO1, ENO2, GLS, GLS2, HK2, PFKFB3, PFKFB, PFKL, PFKM, PFKP, PGK1, SLC2A1 and SLC2A3 and SLC2A5 between the two subtypes in the merged GSE dataset were significantly different, most of which were up-regulated in cluster 2 (Figure 3B). The heat map showed that cluster 2 scored significantly higher in glycolysis, FAO and glutaminolysis pathways than cluster 1 (Figures 3C,D).

### TME characteristics of two metabolic subtypes

**FIGURE 4:**
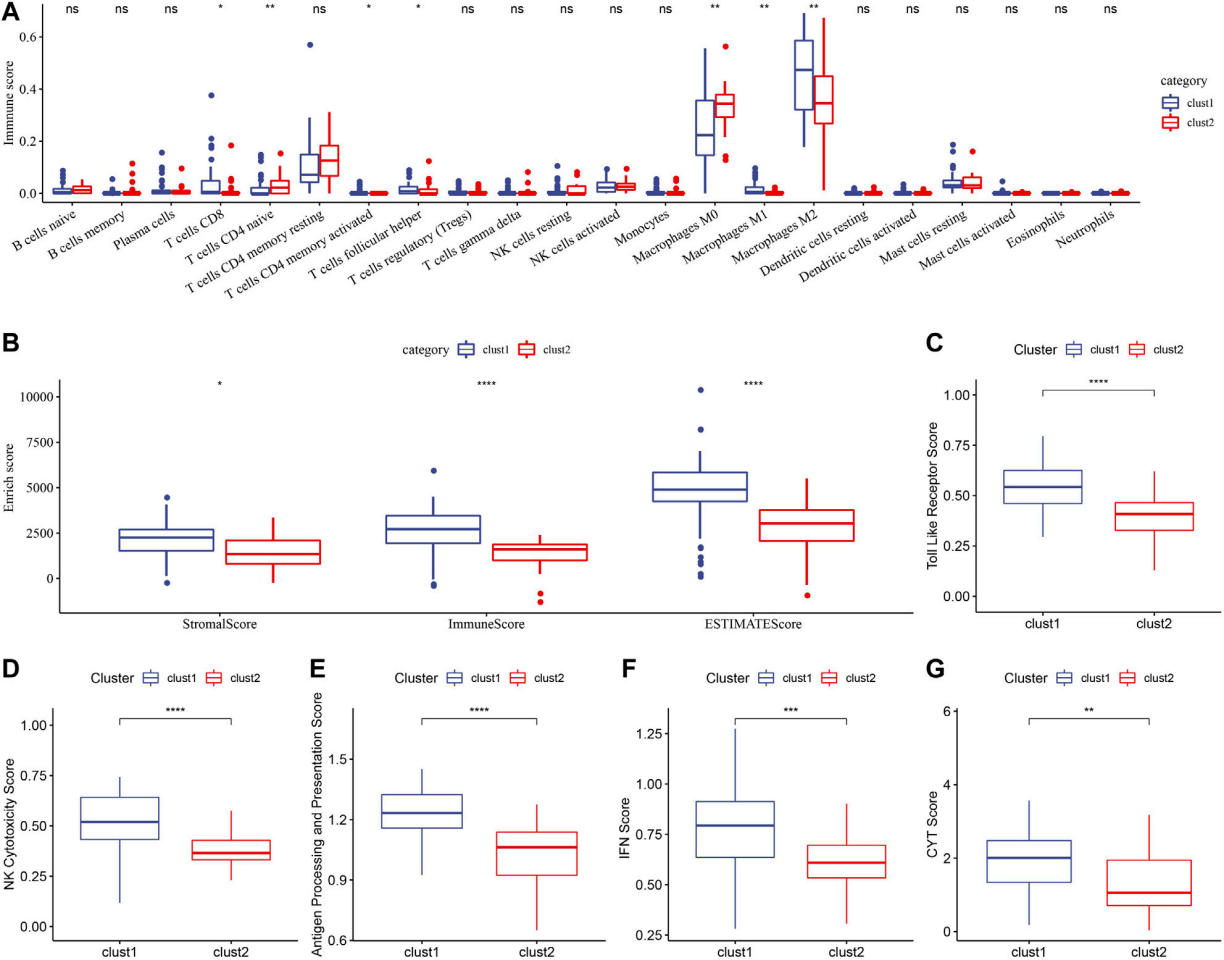
Immune cell infiltration and TME-related scores of two metabolic subtypes. **(A)**: CIBERSORT determined the relative infiltration levels of 22 immune cells between the two metabolic subtypes in Target. **(B)**: ESTIMATE analyzed stromal score and immune score and ESTIMATE score of two metabolic subtypes. **(C)**: Toll-like receptor score comparison between Cluster 1 and cluster 2. **(D)**: NK cytotoxicity score differences between the two metabolic subtypes in Target. **(E)**: The score of antigen processing and presentation in cluster 1 and cluster 2. **(F, G)**: The difference of IFN- γ score and CYT score between the two metabolic subtypes. For this figure, the asterisks idicated the statistical *p* value, *p < 0.05, **p < 0.01, ***p < 0.001, ****p <0.0001.

The TME features of the two metabolic subtypes of osteosarcoma were described by immune cell infiltration, stromal score, immune score, ESTIMATE score, Toll-like receptor score, NK cytotoxicity score, antigen processing and presentation score, IFN- γ score and CYT score. There were significant differences in the infiltration ratio of CD8 T cells, naive CD4 T cells, activated memory CD4 T cells, helper follicular T cells, M0 macrophages and M1 macrophages and M2 macrophages between the two subtypes (Figure 4A). And all the important TME scores evaluated, including stromal score, immune score, ESTIMATE score, toll-like receptor score, NK cytotoxicity score, antigen processing and presentation score, IFN-γ score and CYT score, showed significantly higher levels in cluster 1 than in cluster 2 (Figures 4B–G). Therefore, the TME of cluster1 may show high immune activity.

### PPI of DEGs between metabolic subtypes

**FIGURE 5:**
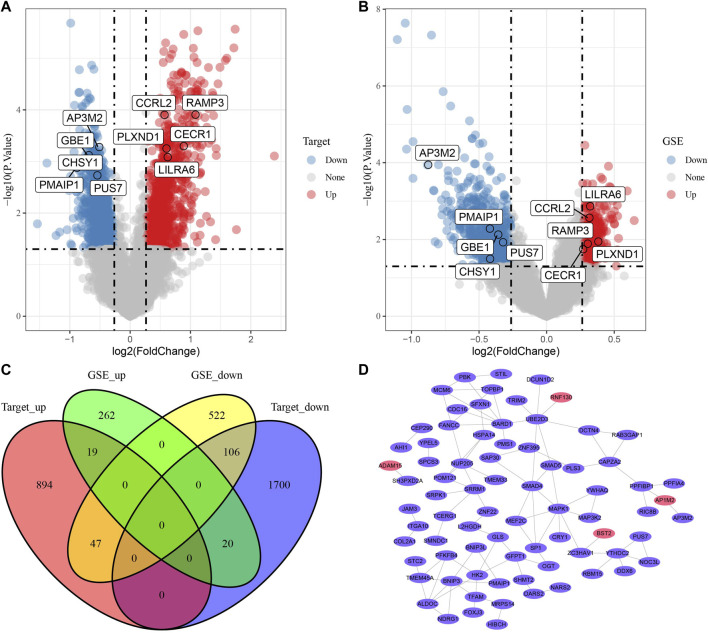
PPI of DEGs between metabolic subtypes. **(A)**: The volcano map of differentially expressed genes in cluster-1 and cluster-2 in Target data, red dots are up-regulated DEGs and blue dots are down-regulated DEGs. **(B)**: Differential expression analysis of two metabolic subtypes in merged GSE data sets. **(C)**: The Venn diagram shows the intersection of the Targets dataset and three GSE queues. **(D)**: The PPI network of 140 genes, the red ellipse represents the up-regulated DEGs and purple ellipse and the down-regulated DEGs.

To distinguish the expression characteristics of the two metabolic subtypes, the differential expression of the two subtypes in Target and merged GSE datasets was analyzed by limma. The results showed that there were 960 up-regulated genes and 1826 down-regulated genes in cluster 2 compared with cluster1 in Target data set (Figure 5A). In the combined GSE dataset, the expression of 301 genes in cluster 2 was significantly higher than that in cluster 1, and the expression of 675 genes in cluster 2 was significantly lower than that in cluster 1 (Figure 5B). The intersection of DEGs in two datasets were taken, 192 common DEGs were obtained (Figure 5C). Based on the score given by STRING, it was considered that there were interactions between genes with score >0.4. Among the 192 DEGs, a total of 140 genes met this condition, and the PPI network between them was shown in Figure 5D. According to degree, we listed 28 genes with the highest status (Supplementary Table S1). The enrichment analysis of GO and KEGG pathway showed that these 140 DEGs were enriched in a wide range of biological processes, involving autophagy and viral carcinogenicity (Supplementary Figure S2).

### Construction and validation of a risk model based on the key DEGs between metabolic subtypes

In Figrue 4C, we obtained 192 DEGs between the two metabolic subtypes, of which 19 were up-regulated and 106 were down-regulated in both datasets. We screened the genes with the greatest impact on the prognosis of OS from these genes with consistent expression in the two datasets to construct a prognostic risk model. First, univariate Cox regression analysis was performed in the Target dataset using “survival” package, and 14 genes were eligible for *p* <0.05 (Supplementary Figure S3A). Then, “glmnet” package performed Least absolute shrinkage and selection operator (Lasso) penalty regression analysis for 14 genes. Lambda = 0.0380 was used to select the best variable and obtain 11 genes (Supplementary Figure S3B). The stepAIC method in “MASS” package finally selected 8 genes from the 11 genes, including STC2, MEF2C, PPFIA4, ITGA10, LILRA6, RNF130, RAB3GAP1, TMEM33. And multivariate Cox regression analysis gave the Cox cofficients of these 8 genes (Supplementary Figure S3C). The value of the product of the expression of each of the eight genes and Cox Cofficient was added to evaluate the risk score of OS samples. Time-dependent receiver operating characteristic curve (ROC) and K-M curve were used to evaluate the prediction accuracy of the risk score model in the Target dataset and the dataset integrating three GSE cohorts. In the former data set, the area under the ROC curve (AUC) value was 0.82, 0.89 and 0.85 at 1 year, 3 years and 5 years, respectively. In addition, a higher risk score indicated a worse prognosis (Supplementary Figure S3D). In the latter dataset, the values of AUC in 1–5 years were 0.65, 0.6, 0.6, 0.7, 0.7, 0.69 respectively. Survival trends in the sample were consistent with those in the Target dataset, with higher risk scores also showing shorter survival times and lower survival rates (Supplementary Figure S3E).

### ScRNA-seq revealed cellular diversity and heterogeneity of osteosarcoma

**FIGURE 6:**
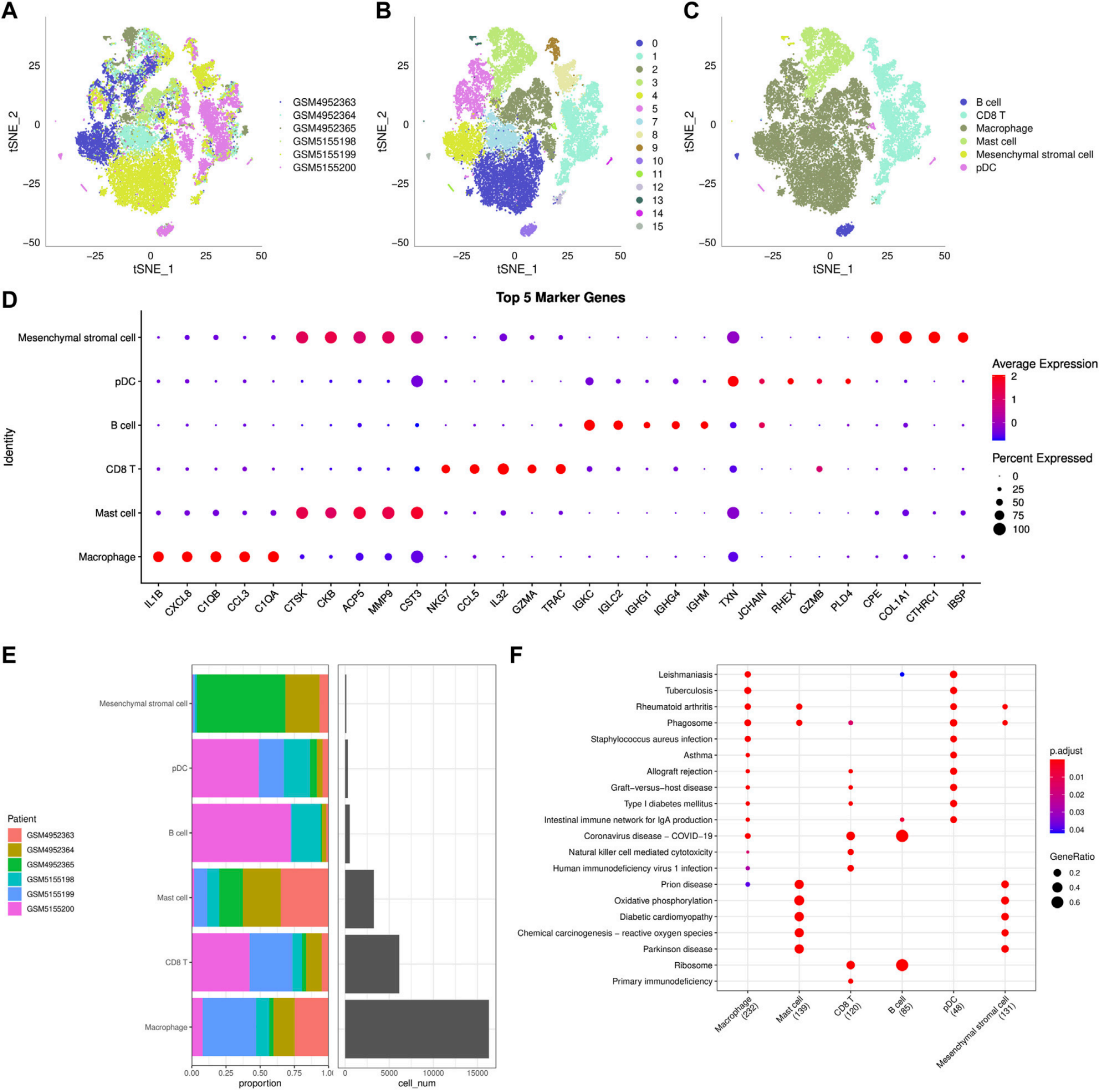
ScRNA-seq reveals cellular diversity and heterogeneity of osteosarcoma **(A–C)**: t-SNE plot of all the single cells, with each color coded for sample source **(A)**, cluster **(B)**, and immune cell type **(C)**. **(D)**: The bubble diagram showed the average expression of top5 marker genes in 6 kinds of immune cells. **(E)**: The distribution of 6 types of immune cells in each sample. **(F)**: KEGG enrichment analysis bubble map for the marker genes of each type of immune cells.

Transcripts of 44,516 cells were obtained by quality control of 50,174 cells from six tumor tissues in the GSE162454 dataset (Supplementary Figure S4B–4C). Correlation analysis showed that there was a significant positive correlation between sequencing depth and the number of mRNA, but no significant correlation with mitochondrial gene sequences (Supplementary Figure S4A). Preliminary PCA dimensionality reduction identified 35 clusters of 44,516 cells. A further 28608 immune cells were recognized from 44516 cells using the marker PTPRC (CD45) (Supplementary Figure S4D). PCA was performed on 28,608 immune cells identified by highly variable genes (Supplementary Figure S4E). After t-SNE analysis, 28608 immune cells from 6 osteosarcoma samples were classified into 16 clusters. It is worth noting that cluster 6 does not belong to immune cells (Figures 6A,B). Based on the annotation of 15 clusters of characteristic genes of immune cells, 6 types of immune cells were obtained: B cell (cluster 10, 15), CD 8 T cells (cluster 1, 8, 9, 12), macrophage (cluster 0, 2, 4, 5, 7), mast cell (cluster 3), mesenchymal stromal cell (cluster 13), plasmacytoid dendritic cells (pDC, cluster 11, 14) (Figure 6C, Supplementary Figure S5). Then the marker genes of each type of immune cells were identified by FindMarkers (Figure 6D). It also showed the distribution of six types of immune cells in each sample, and the proportion of six kinds of immune cells in different samples was different, indicating that there was heterogeneity in the distribution of immune cells among patients (Figure 6E). KEGG enrichment analysis based on the marker genes of each type of immune cells showed that immune-related pathways, such as rheumatoid arthritis, allograft rejection and intestinal immune network for IgA production, were significantly up-regulated in macrophage and pDC. The enriched signal pathways and their trends in mast cell and mesenchymal stromal cell were the same. In addition, coronavirus disease-COVID-19 and ribosome were significantly activated in CD 8 T cell and B cell. Therefore, six types of immune cells regulated a wide range of biological functions.

### Composition and metabolism of malignant cells

**FIGURE 7:**
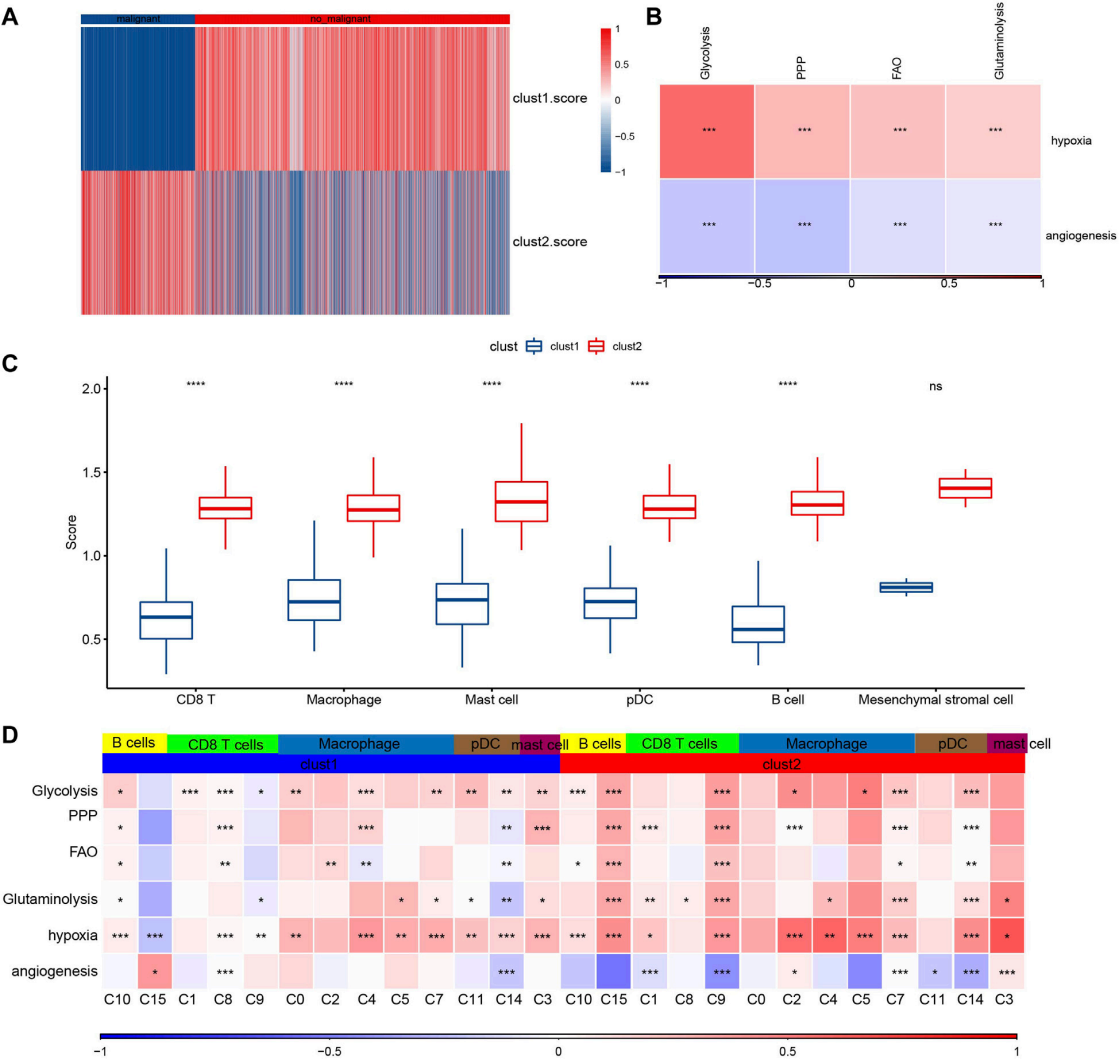
Composition and metabolism of malignant cells. **(A)**: The heat map shows the proportion of malignant and non-malignant cells between clusters 1 and cluster 2. **(B)**: The correlation between hypoxia score/angiogenesis score and four metabolic scores for malignant cells. **(C)**: The difference between the enrichment scores of six kinds of immune cells in cluster 2 and in cluster 1. **(D)**: Pearson correlation analysis showed the relation between 5 kinds of immune cells/15 subgroups in cluster 1 and cluster 2 and 4 metabolic patterns, hypoxia and angiogenesis, respectively. For this figure, the asterisks idicated the statistical *p* value, *p < 0.05, **p < 0.01, ***p < 0.001, ****p <0.0001.

To further verify the malignant traits of the two metabolic subtypes obtained by bulk RNA-seq analysis, copycat (Dietz et al., 2021) was used to calculate the large-scale chromosomal copy number variation (CNV) in each fine cell type based on the scRNA-seq data of six kinds of immune cells, so as to distinguish malignant cells from non-malignant cells in each sample. There was an obvious difference in the proportion of malignant cells and non-malignant cells between cluster 1 and cluster 2. The proportion of non-malignant cells in cluster 1 was much higher than that in malignant cells, while the proportion of non-malignant cells in Cluster 2 was the opposite (Figure 7A). Next, the composition of 7617 malignant cells were analyzed. Of the 7617 malignant cells, CD8 T cell accounted for the majority (Supplementary Table S2). The genes in hallmark hypoxia pathway were selected and ssGSEA was used to calculate the hypoxia score of malignant cells. According to the same method, the angiogenesis score of malignant cells was obtained according to the expression of 24 angiogenic genes (Masiero et al., 2013). Pearson correlation analysis showed that there was a significant positive correlation between hypoxia score and the expression of HIF-1α, the main molecular mediator of hypoxia adaptation in tumor cells, with correlation coefficient R = 0.313 (Supplementary Figure S6). And hypoxia score was also significantly positively correlated with the four metabolic scores, while angiogenesis score was significantly negatively correlated with the four metabolic scores. (Figure 7B). The enrichment scores of all six immune cells in cluster 2 were higher than those in cluster 1, and except for mesenchymal stromal cell, the enrichment scores of the other five immune cells showed significant differences between the two subtypes (Figure 7C). Finally, comprehensive Pearson correlation analysis showed the relation between 5 kinds of immune cells/15 subgroups in cluster 1 and cluster 2 and 4 metabolic patterns, hypoxia and angiogenesis, respectively (Figure 7D).

## Discussion

Cancer metabolism, as with all processes in life, is comprised of both genetic and environmental components (Bose et al., 2020). Cancer metabolism has gained substantial research interest over recent years (Weber, 2016). We are just beginning to understand the heterogeneity of metabolic phenotypes. It is likely that metabolic phenotypes may differ due to several factors: primary or metastatic tumor, tumor location, tumor microenvironment and mutation (Kubicka et al., 2021). Multiple metabolic subtypes related to the prognosis of different cancers have been reported (Liu et al., 2021a; Liu et al., 2021b; Gao et al., 2021; Lin et al., 2021). Different from these studies, we not only used metabolism-related genes to define OS subtypes to characterize inter-tumor heterogeneity, but also revealed intra-tumor heterogeneity through bulkRNA-seq and scRNA-seq data analysis, and explored the relationship between metabolism and tumor malignancy and TME.

Based on genes associated with four abnormal metabolic phenotypes of cancer, we revealed two metabolic subtypes that showed different prognosis. The enrichment scores of glycolysis, FAO and glutaminolysis pathways in cluster 2 with worse prognosis were significantly higher than those in cluster 1, indicating that these metabolisms in cluster 2 were more active. OSs, grow in the bone microenvironment, a very specialized, complex, and highly dynamic environment composed of bone cells (osteoclasts, osteoblasts, osteocytes), stromal cells (MSCs, fibroblasts), vascular cells (endothelial cells and pericytes), immune cells (macrophages, lymphocytes), and a mineralized extracellular matrix (ECM) (Corre et al., 2020). The core characteristics of establishing metabolic phenotypes include unfavorable TME (Kubicka et al., 2021). In this paper, the TME of the identified metabolic subtypes was detected, and it was found that the TME of the two metabolic subtypes had significantly different characteristics. Cluster 2 showed low levels of TME, stromal score, immune score, ETIMATE score, toll-like receptor, NK cytotoxicity, antigen processing and presentation and IFN- γ and CYT scores. These results indirectly support the reliability of the identified metabolic subtypes and their association with TME.

We constructed a 8-gene siganture based on the DEGs between the two metabolic subtypes with consistent expression trend in two datasets, and the effects of some of these genes on tumor development have been explored and reported. One study has found that STC2 promotes the development and progression of OS by enhancing glycolysis (Yu et al., 2021). The regulatory effect of MEF2C on a variety of malignancies has been widely studied, including its role in the regulation of iron death in meningioma (Bao et al., 2021) and its involvement in brain metastases of human breast cancer (Galego et al., 2021). High expression of PPFIA4 is associated with poor prognosis in colon cancer patients and promotes cancer cell metastasis by enhancing tumor glycolysis (Huang et al., 2021). TMEM33 expression is increased in cervical cancer and can be used as an independent prognostic marker (Chen et al., 2022). The effect of the combination of these genes and the remaining four genes on osteosarcoma is unknown. In this study, the survival and ROC curves of 8-gene siganture in the two datasets showed that the 8-gene siganture had moderate predictive performance for the prognosis of OS.

Immune cells are the key components of TME. There is intra-tumor heterogeneity among immune cells in OS(26). We also analyzed the immune cells of OS at the single cell level and identified 15 single immune clusters, which were annotated to B cell, CD 8 T cells, macrophage, mast cell, mesenchymal stromal cell, plasmacytoid dendritic cells. In OS, the distribution of six kinds of immune cells was heterogeneous and regulated a wide range of biological pathways.

Tumors are composed of complex environments of malignant and non-malignant cell types with different metabolic preferences (Lasche et al., 2020). Here, we distinguish between malignant and non-malignant cells in all immune cells by calculating the large-scale CNV in each cell type. CD8 T cell accounted for a high proportion of malignant cells. TME, determined by abnormal metabolism of cancer cells, is characterized by the hypoxia and induction of angiogenesis (Roma-Rodrigues et al., 2019). In this study, the scores of glycolysis, PPP, FAO and glutaminolysis of malignant cells were positively correlated with hypoxia scores and negatively correlated with angiogenesis score. The results also confirmed the relationship between the metabolism of malignant cells and TME.

Overall, we defined two molecular subtypes of OS with unique metabolic patterns and TME based on metabolism-related genes, and constructed a 8-gene siganture based on the DEGs between the two metabolic subtypes with consistent expression trend in two datasets, as well as revealed 16 separate clusters and 6 immune cell types based on bulkRNA-seq and scRNA-seq. We focus on the malignant cells of the immune cell group, which were characterized by hypoxia and exuberant angiogenesis and were closely related to metabolism. Our work provides important insights into the malignant and immune cell maps and their effects on metabolic patterns in OS.

## Data Availability

The datasets presented in this study can be found in online repositories. The names of the repository/repositories and accession number(s) can be found in the article/Supplementary Material.
