# Supplementary figures and images for: Combining bulk RNA-sequencing and single-cell RNA-sequencing data to reveal the immune microenvironment and metabolic pattern of osteosarcoma
Source: Front Genet. 2022 Oct 19;13:976990. doi: 10.3389/fgene.2022.976990 (PMC9626532; doi:10.3389/fgene.2022.976990)

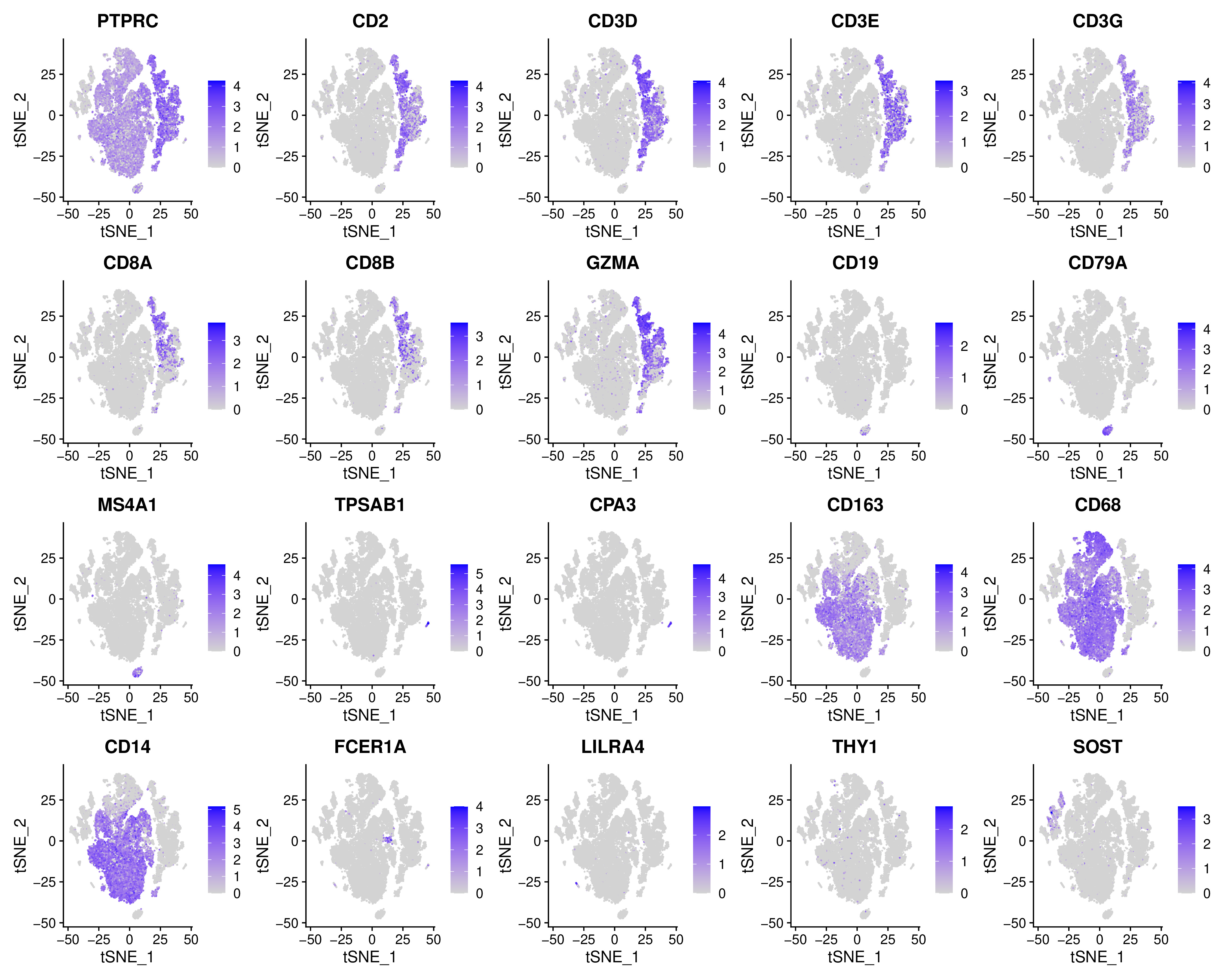

Supplement: Supplementary file 1 [file Image5.jpg]

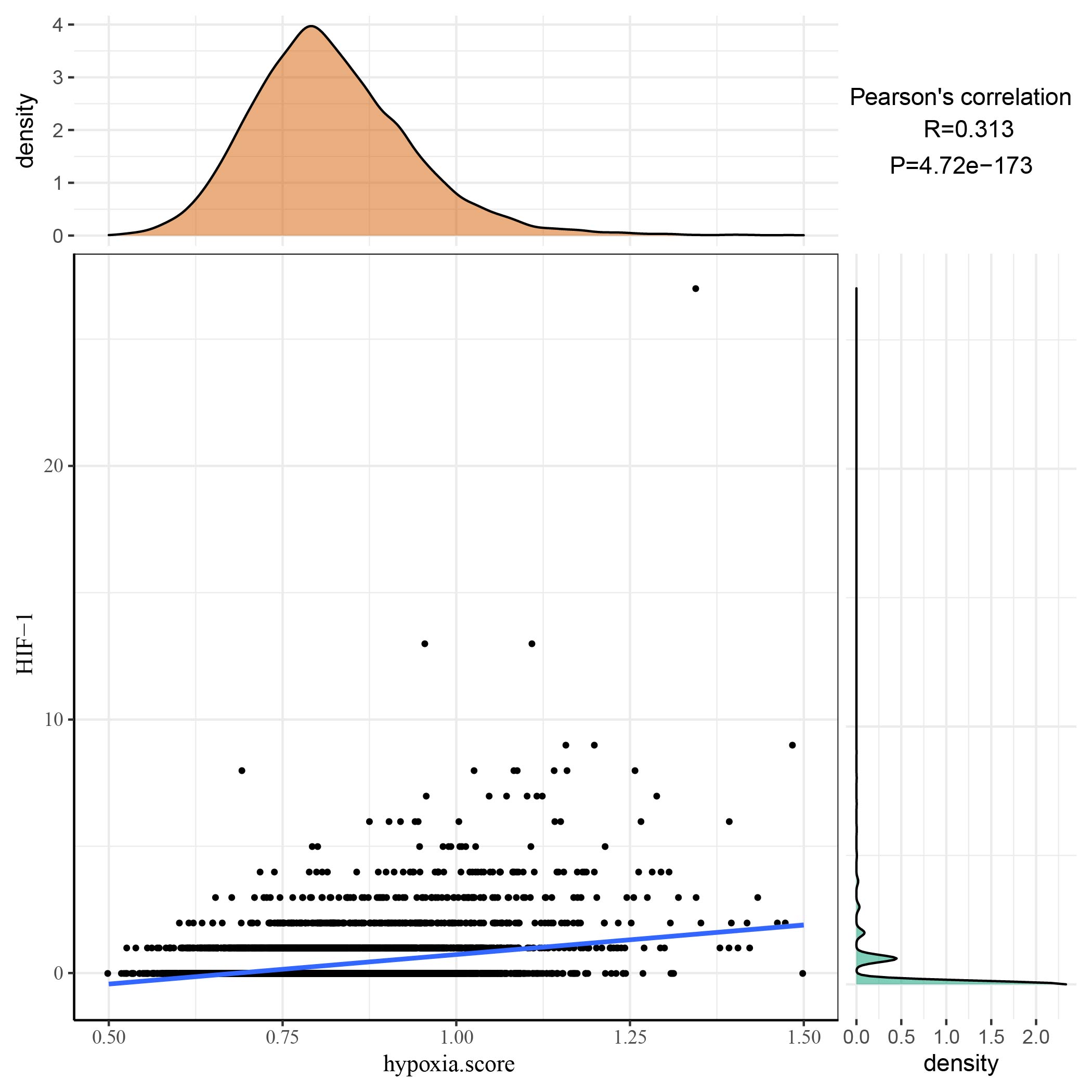

Supplement: Supplementary file 2 [file Image6.jpg]

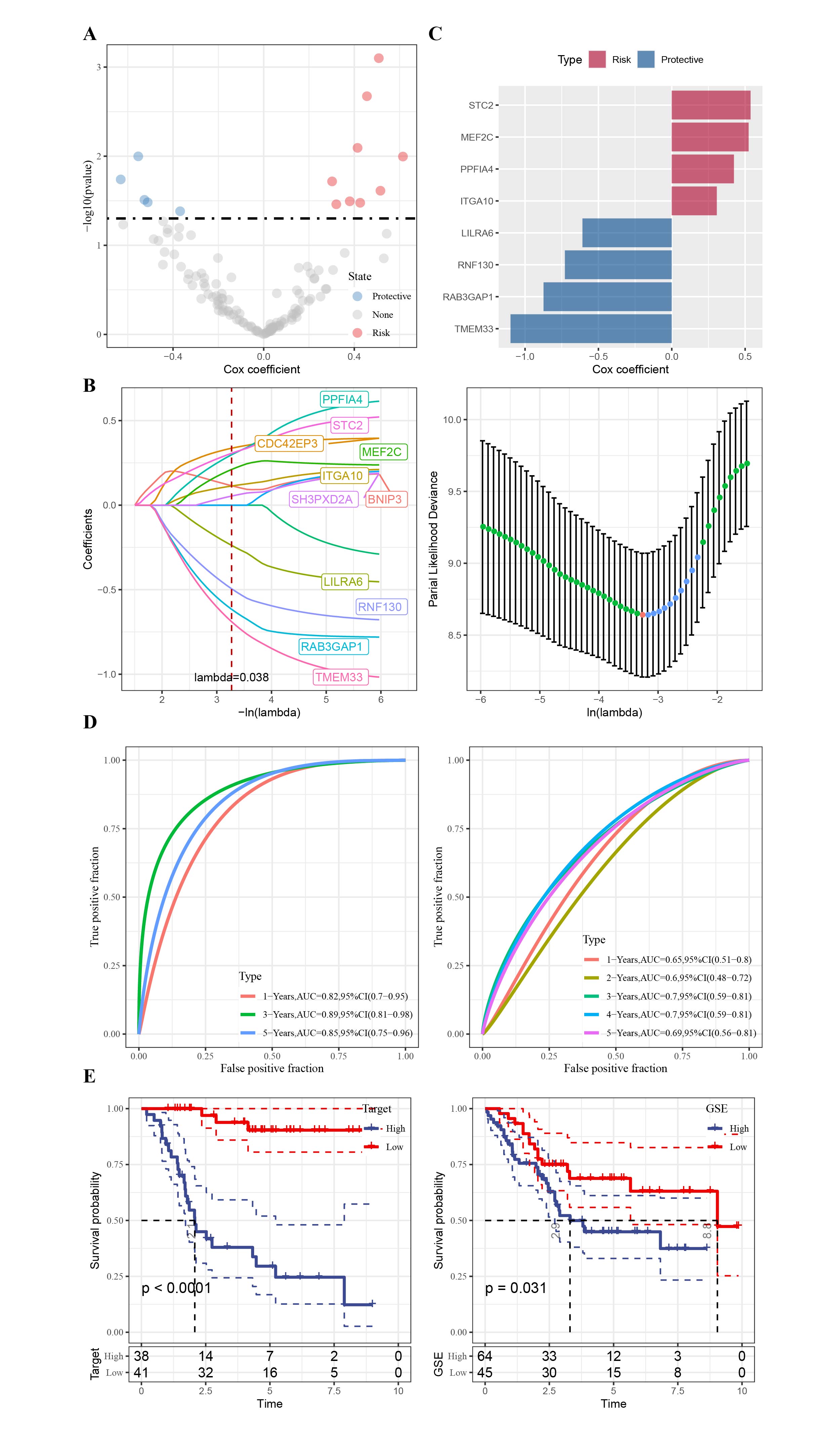

Supplement: Supplementary file 3 [file Image3.jpg]

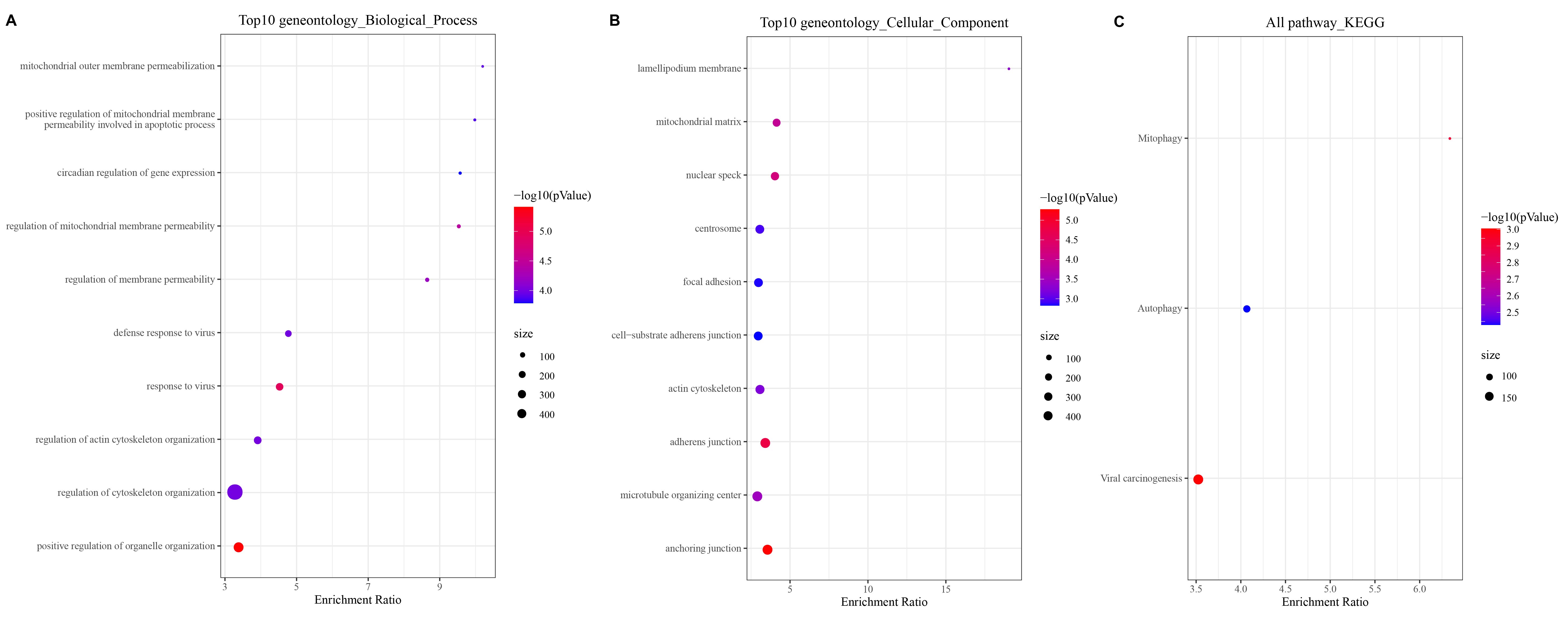

Supplement: Supplementary file 4 [file Image2.jpg]

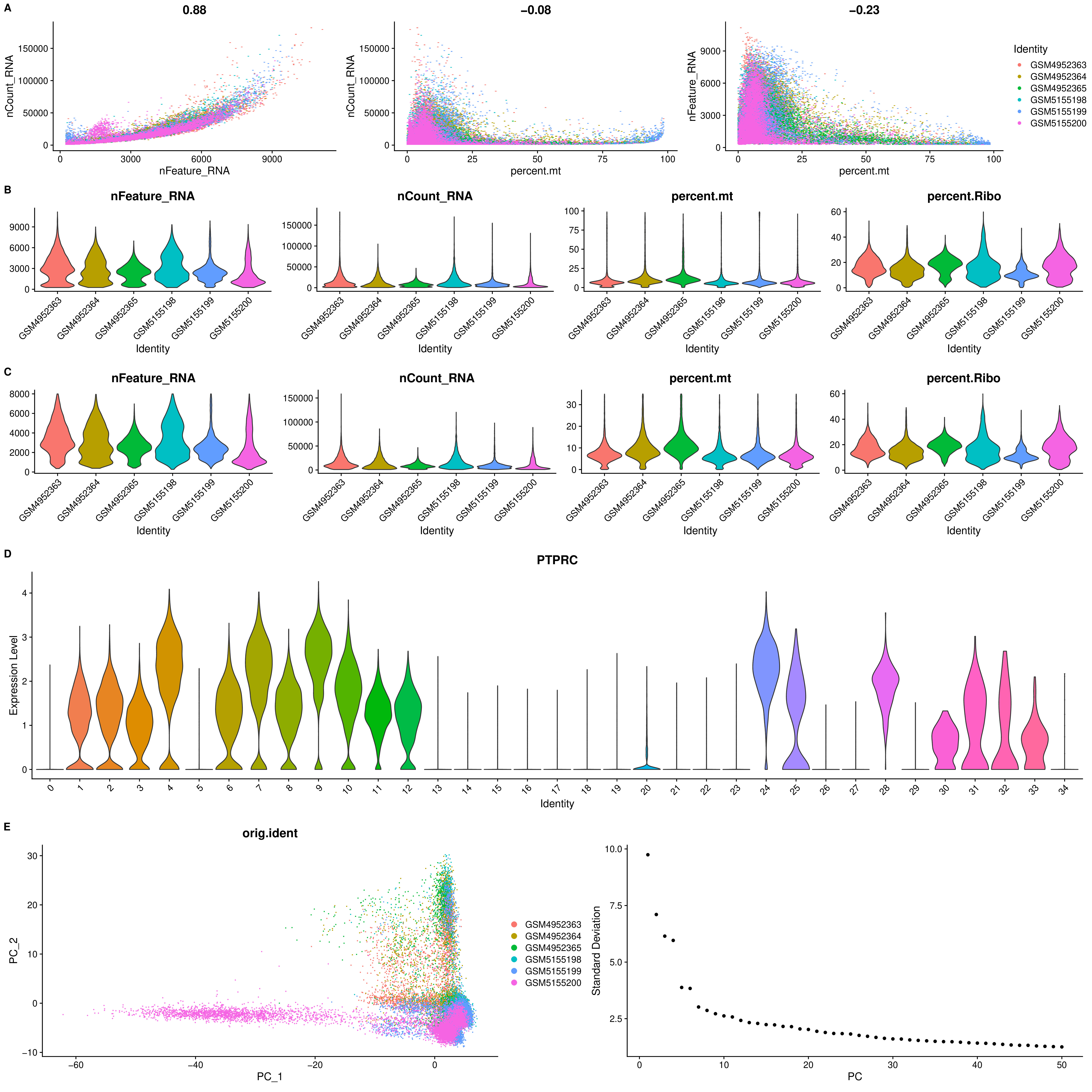

Supplement: Supplementary file 7 [file Image4.jpg]

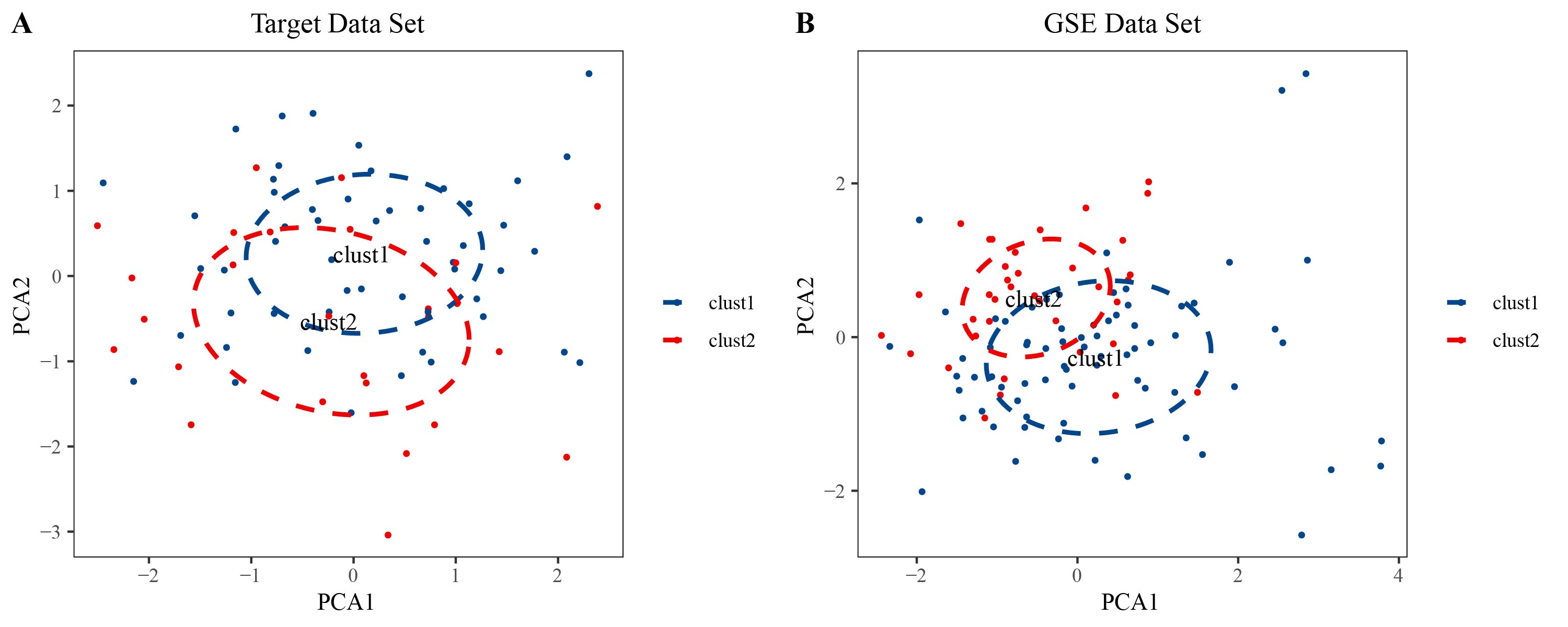

Supplement: Supplementary file 8 [file Image1.jpg]
