# Supplementary material for: Combining bulk RNA-sequencing and single-cell RNA-sequencing data to reveal the immune microenvironment and metabolic pattern of osteosarcoma
Source: Front Genet. 2022 Oct 19;13:976990. doi: 10.3389/fgene.2022.976990 (PMC9626532; doi:10.3389/fgene.2022.976990)
Supplement: Supplementary file 5 [file Table1.DOCX]

**Table S1 28 genes with the highest degree in PPI network**

| name | Degree |
| --- | --- |
| MAPK1 | 11 |
| BNIP3 | 9 |
| BARD1 | 9 |
| CFL1 | 8 |
| UBE2D3 | 8 |
| BECN1 | 8 |
| CHAF1A | 7 |
| IRF3 | 7 |
| SMAD4 | 7 |
| LILRB2 | 6 |
| IKBKG | 6 |
| CD300C | 6 |
| TFAM | 6 |
| CAPZA2 | 6 |
| YTHDC2 | 6 |
| SP1 | 6 |
| ALDOC | 5 |
| HK2 | 5 |
| TOPBP1 | 5 |
| PBK | 5 |
| MCM6 | 5 |
| BNIP3L | 5 |
| PLS3 | 5 |
| LCP1 | 5 |
| ITGAL | 5 |
| FLNA | 5 |
| NUP205 | 5 |
| SRRM1 | 5 |
