# Supplementary material for: Combining bulk RNA-sequencing and single-cell RNA-sequencing data to reveal the immune microenvironment and metabolic pattern of osteosarcoma
Source: Front Genet. 2022 Oct 19;13:976990. doi: 10.3389/fgene.2022.976990 (PMC9626532; doi:10.3389/fgene.2022.976990)
Supplement: Supplementary file 6 [file Table2.DOCX]

**Table S2 Composition of 7617 malignant cells**

|  | C0 | C1 | C2 | C3 | C4 | C5 | C6 | C7 | C8 | C9 | C10 | C11 | C12 | C13 | C14 | C15 |
| --- | --- | --- | --- | --- | --- | --- | --- | --- | --- | --- | --- | --- | --- | --- | --- | --- |
| B cell | 0 | 0 | 0 | 0 | 0 | 0 | 0 | 0 | 0 | 0 | 399 | 0 | 0 | 0 | 0 | 77 |
| CD8 T cell | 0 | 4338 | 0 | 0 | 0 | 0 | 0 | 0 | 1041 | 365 | 0 | 0 | 1 | 0 | 0 | 0 |
| Macrophage | 188 | 0 | 452 | 0 | 67 | 78 | 0 | 143 | 0 | 0 | 0 | 0 | 0 | 0 | 0 | 0 |
| Mast cell | 0 | 0 | 0 | 281 | 0 | 0 | 0 | 0 | 0 | 0 | 0 | 0 | 0 | 0 | 0 | 0 |
| Mesenchymal stromal cell | 0 | 0 | 0 | 0 | 0 | 0 | 0 | 0 | 0 | 0 | 0 | 0 | 0 | 2 | 0 | 0 |
| pDC | 0 | 0 | 0 | 0 | 0 | 0 | 0 | 0 | 0 | 0 | 0 | 123 | 0 | 0 | 62 | 0 |
|  |  |  |  |  |  |  |  |  |  |  |  |  |  |  |  |  |
