# Supplementary material for: Combining bulk RNA-sequencing and single-cell RNA-sequencing data to reveal the immune microenvironment and metabolic pattern of osteosarcoma
Source: Front Genet. 2022 Oct 19;13:976990. doi: 10.3389/fgene.2022.976990 (PMC9626532; doi:10.3389/fgene.2022.976990)
Supplement: Supplementary file 9 [file DataSheet1.docx]

Supplementary Figure description

Fig S1 PCA based on the total mRNA expression profile of Target dataset (A) and GSE merged dataset (B).

Fig S2 Pathway enrichment analysis of 140 genes

A: GO Biological process analysis of 140 genes showed that there were 10 items in the first. B: The top 10 GO Cellular Components enriched by 140 genes. C: 10 KEGG pathways with the highest enrichment degree of 140 genes.

Fig S3 Construction and validation of a risk model based on the key DEGs between metabolic subtypes

A: The volcano plot shows the prognostic risk genes and protection genes in the 125 genes. B: Lasso penalty regression analysis for 14 genes. C: Multivariate Cox regression analysis gave Cox cofficients of 8 genes. D: The ROC curve and K-M curve for OS samples in Target dataset. E: The ROC curve and K-M curve for OS samples in the dataset integrating three GSE cohorts.

Fig S4 ScRNA-seq data processing and analysis of osteosarcoma

A: Correlation analysis showed the correlation between sequencing depth and the number of mRNA, the correlation between sequencing depth and mitochondrial gene sequences, the correlation between the number of mRNA and mitochondrial gene sequences. B: The number of original expressed genes, the content of total count distribution, mitochondrial gene and rRNA in osteosarcoma samples. C: After quality control, the number of expressed genes, total count distribution, mitochondrial gene and rRNA content in each sample. D: The expression level of CD45 in 35 clusters of 44516 cells. E: PCA dimension reduction analysis of 28608 immune cells.

Fig S5 t-SNE diagram of representative marker genes expression

Fig S6 Pearson correlation analysis hsowed the relationship between HIF-1α expression and hypoxia score.
